# CD45-Positive Small Lymphocyte-Like Myeloma with *IGH::CCND1* Fusion and *TP53* Mutations

**DOI:** 10.1007/s12288-025-02063-2

**Published:** 2025-06-07

**Authors:** Ke Xu, Evan Vitsaras, Anna Childerhouse, Temenuzhka Boneva, Elisabeth Nacheva, Rajeev Gupta

**Affiliations:** 1 Department of Haematology, University College London Hospitals NHS Foundation Trust, University College London, London, UK; 2 Specialist Integrated Haematology Malignancy Diagnostic Service, Health Services Laboratories, University College London Hospitals NHS Foundation Trust, University College London, London, UK; 3 Department of Histopathology, University College London Hospitals NHS Foundation Trust, University College London, London, UK; 4 UCL School of Life and Medical Sciences, London, UK

**Fig. 1:**
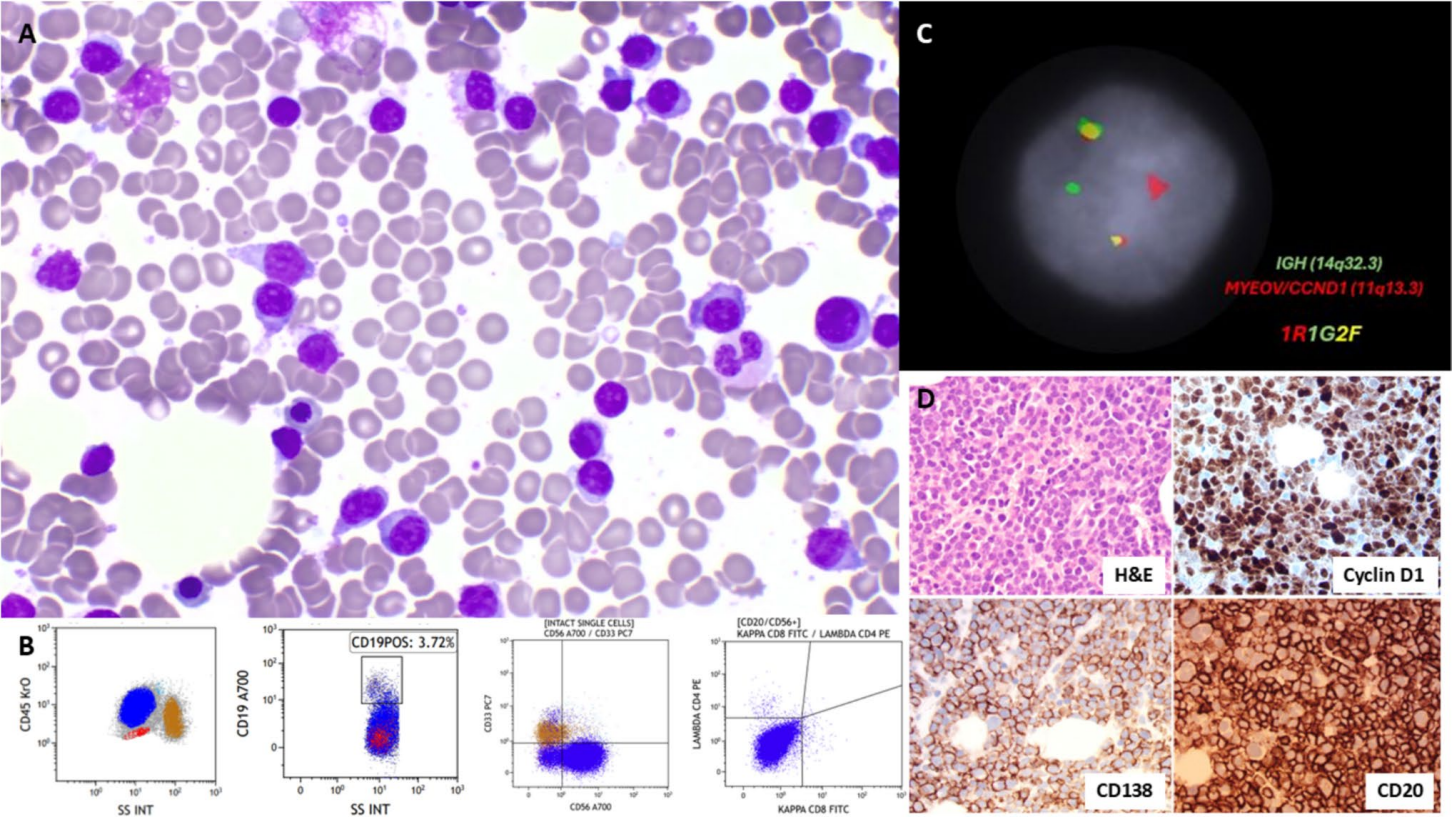
**A** Bone marrow aspirate (Giemsa May-Grunwald stain × 100 objective). **B** Immunophenotyping (blue colour population). **C** FISH showing *IGH::CCDN1* (DAPI staining, × 100 objective, Cytocell probes). **D** Bone marrow trephine biopsy (× 40 objective) was positive for CD138, cyclin D1, CD20

A 52-year-old male presented with hypercalcaemia and acute kidney injury. The serum-free kappa chain was 956 mg/L, and the K:L ratio was 119; paraprotein was not detected. A bone marrow aspirate showed excess small lymphocytoid cells (Fig. 1A). They were positive for CD45 and CD56 and negative for CD34, CD19, CD2, CD5, CD7, CD4, CD8, CD33, CD117, CD15, CD13, CD57, surface Ig, MPO, and cTdT (Fig. 1B). Small lymphocyte-like myeloma was suspected, but the addition CD38, CD138 and cytoplasmic Ig could not be performed on flow cytometry due to sample limitation. Targeted CD138 cell FISH showed *IGH::CCND1* fusion (Fig. 1C). Trephine IHC showed CD138+, CD56+, cyclin D1+, CD20+, kappa-restricted small plasma cells (Fig. 1D), confirming the diagnosis of myeloma. Lymphoid NGS identified pathogenic *TP53* p.Glu285Lys (VAF 62%, COSM10722) and *TP53* p.Val272Leu (VAF 19%, COSM10859) variant. The patient had a short response to multiple lines of treatment, refractory to elranatamab and had only two years of overall survival.

Small-lymphocytes-like plasma cell myeloma could mimic mature B-cell lymphoma with or without plasmacytic differentiation. Adding plasma cell markers in the flow panel for lymphoma and adding cytoplasmic kappa/lambda stain in surface Ig-negative cases could help better detect atypical myeloma cases. Small-lymphocytes-like plasma cell myeloma is usually a standard risk with frequent CD20+ and t(11;14) [1]. Detecting pathogenic *TP53* variants makes this case high-risk. Gonsalves reported that CD45 expression is an independent poor risk factor of myeloma overall survival in the era of novel treatment agents [2]. This case highlighted the importance of incorporating flow cytometry, molecular testing for diagnosis and full-risk stratification.
